# Safety and Efficacy of Megakaryocytes Induced from Hematopoietic Stem Cells in Murine and Nonhuman Primate Models

**DOI:** 10.5966/sctm.2016-0224

**Published:** 2016-10-07

**Authors:** Xin Guan, Meng Qin, Yu Zhang, Yanan Wang, Bin Shen, Zhihua Ren, Xinxin Ding, Wei Dai, Yongping Jiang

**Affiliations:** ^1^ Biopharmaceutical R&D Center, Chinese Academy of Medical Sciences & Peking Union Medical College, Suzhou, People's Republic of China; ^2^ Biopharmagen Corp., Suzhou, People's Republic of China; ^3^ Department of Laboratory Diagnosis, Suzhou Municipal Hospital Affiliated Nanjing Medical University, Suzhou, People's Republic of China; ^4^ College of Nanoscale Science, SUNY Polytechnic Institute, Albany, New York, USA; ^5^ Department of Environmental Medicine, New York University Langone Medical Center, Tuxedo, New York, USA

**Keywords:** Megakaryocytes, Thrombocytopenia, Hematopoietic stem cells, Expansion and differentiation, Transplantation, Nonhuman primates

## Abstract

Because of a lack of platelet supply and a U.S. Food and Drug Administration‐approved platelet growth factor, megakaryocytes have emerged as an effective substitute for alleviating thrombocytopenia. Here, we report the development of an efficient two‐stage culture system that is free of stroma, animal components, and genetic manipulations for the production of functional megakaryocytes from hematopoietic stem cells. Safety and functional studies were performed in murine and nonhuman primate models. One human cryopreserved cord blood CD34^+^ cell could be induced ex vivo to produce up to 1.0 × 10^4^ megakaryocytes that included CD41a^+^ and CD42b^+^ cells at 82.4% ± 6.1% and 73.3% ± 8.5% (mean ± SD), respectively, yielding approximately 650‐fold higher cell numbers than reported previously. Induced human megakaryocytic cells were capable of engrafting and producing functional platelets in the murine xenotransplantation model. In the nonhuman primate model, transplantation of primate megakaryocytic progenitors increased platelet count nadir and enhanced hemostatic function with no adverse effects. In addition, primate platelets were released in vivo as early as 3 hours after transplantation with autologous or allogeneic mature megakaryocytes and lasted for more than 48 hours. These results strongly suggest that large‐scale induction of functional megakaryocytic cells is applicable for treating thrombocytopenic blood diseases in the clinic. Stem Cells Translational Medicine *2017;6:897–909*

**Significance Statement**

Because of platelet shortage and a lack of U.S. Food and Drug Administration‐approved platelet growth factor, megakaryocytes have been regarded as an effective substitute for platelets. This study showed an efficient method to generate functional megakaryocytes from CD34^+^ cells. The amount of megakaryocytes derived from one cord blood unit (5 million CD34^+^ cells) could, in theory, be used for treatment for approximately 30 patients. Moreover, autologous and allogeneic transplantation studies in nonhuman primates strongly suggest the safety and efficiency of these induced megakaryocytes. This investigation may serve as a novel way to alleviate thrombocytopenia in the clinic.

## Introduction

Platelets play a crucial role in physiological hemostasis, and reduced platelet numbers lead to coagulation defects and uncontrollable bleeding [1, 2, 3]. Severe and prolonged thrombocytopenia frequently occurs in patients receiving high‐dose chemotherapy or in those who undergo hematopoietic stem cell (HSC) transplantation [4, 5]. At present, platelet transfusion is the primary method for protecting these patients from thrombocytopenic bleeding. However, platelet transfusion therapy in the clinic remains limited because of a lack of supply, short shelf‐life, and potential refractoriness to repeated transfusions [6].

In general, the delay in platelet recovery after chemotherapy or autologous/allogeneic HSC transplantation may be partly associated with an insufficiency of megakaryocyte (MK)‐committed cells [7]. Therefore, one strategy to accelerate platelet recovery is to implant a sufficient number of MK‐committed repopulating cells until hematopoietic reconstitution occurs in the patient [8]. It has been reported that a higher proportion of megakaryocytic progenitors (MKPs) in transplanted grafts positively affects the platelet recovery [9, 10]. However, a sufficient number of ex vivo expanded MKs is difficult to obtain for clinical applications. Although some research groups have obtained immortalized MKP cell lines from induced pluripotent stem (iPS) cells [11, 12], their safety remains questionable, given that these cell lines were obtained from virus‐transduced iPS cells. Alternatively, human cord blood (hCB)‐derived stem cells have emerged as an excellent source for HSC transplantation because of their ease of collection, their greater proliferation rate, and less stringent requirements for HLA matching [13, 14, 15].

A major hurdle in generating MKs ex vivo is low purity of induced MKs after CD34^+^ cell expansion. Several groups have tried to produce MKs by a multistep culture system in which CD34^+^ cells were not specifically expanded, thereby producing mature cells of various lineages [16, 17]. In addition, most in vivo studies in the past were simply related to kinetics and engraftment in murine models, which are limited by the relatively short life span of mice and species differences between humans and mice. Nonhuman primates are considered to be valuable mammalian models for cell transplantation studies because the regulation of hematopoiesis in these models is physiologically relevant to humans [18, 19].

Here, we demonstrate a stem cell expansion and differentiation platform that can generate MKPs and mature MKs ex vivo on a large scale. We also evaluated long‐term safety and platelet‐releasing efficacy of induced megakaryocytes in murine and nonhuman primate models.

## Materials and Methods

### Cytokines, Antibodies, and Reagents

Recombinant human stem cell factor (SCF), Flt‐3 ligand (Flt‐3L), interleukin (IL)‐3, granulocyte colony‐stimulating factor (G‐CSF), thrombopoietin (TPO), granulocyte‐macrophage colony‐stimulating factor (GM‐CSF), and insulin were purchased from Biopharmagen Corp. (Suzhou, China, http://www.biopharmagen.com/). IL‐6 was purchased from PeproTech Inc. (Rocky Hill, NJ; http://www.peprotech.com). StemRegenin 1 (SR1) was from Selleck Chemicals (Houston, TX, http://www.selleckchem.com/). Fluorescein isothiocyanate (FITC)‐conjugated anti‐CD61 antibody, FITC‐conjugated anti‐CD41a antibody, phycoerythrin (PE)‐conjugated anti‐CD34 antibody, allophycocyanin (APC)‐ conjugated anti‐CD42b antibody, PE‐conjugated anti‐P‐selectin (CD62P) antibody, APC‐conjugated anti‐CD15 antibody, and PE‐conjugated anti‐CD19 antibody were purchased from BD Biosciences (San Diego, CA, http://www.bdbiosciences.com). Putrescine, selenium, transferrin, and ADP were from Sigma‐Aldrich (St. Louis, MO, http://www.sigmaaldrich.com). B‐27 supplements were from Thermo Fisher Scientific Life Sciences (Waltham, MA, http://www.thermofisher.com).

### Animal Care and Ethics Statements

All research involving animals was conducted according to relevant national and international guidelines. Male NOD/SCID mice at 5–6 weeks of age were purchased from the Shanghai Laboraroty Animal Co. (SLAC, Shanghai, China, http://www.slaccas.com/). The mouse experiment protocols were approved by the Institutional Animal Care and Use Committees of Soochow University (IACUC Permit Number SYXK [Su] 2012‐0045).

Cynomolgus macaques were from the Medical Primate Research Center of the Institute of Medical Biology, Chinese Academy of Medical Sciences, and housed and bred according to the guidelines. The experimental protocols were reviewed and approved by the Yunnan Province Experimental Animal Management Association (Permit Number SYXK‐YN 2010‐0009) and the Experimental Animal Ethic Committee of the Institute, which complied with the humane regulations of replacement, refinement, and reduction [20].

### Collection of CD34^+^ Cells

Fresh hCB samples and cryopreserved hCB nucleated cells were obtained from Suzhou Municipal Hospital (Suzhou, China) after written informed consent that was approved by the Suzhou Municipal Hospital Ethics Committee and Research Ethics Advisory Committee. Mononuclear cells were isolated by a Ficoll‐Paque (GE Healthcare, Marlborough, MA, http://www.gehealthcare.com) density gradient centrifugation [21]. CD34^+^ cells were enriched from mononuclear cells by magnetic cell sorting using a MACS Direct CD34 MicroBead Kit (Miltenyi Biotec, Amsterdam, The Netherlands, http://www.miltenyibiotec.com) as per the manufacturer’s instructions. Enriched CD34^+^ cells were confirmed by flow cytometry (BD Biosciences) after staining with a PE‐conjugated anti‐CD34 antibody with a purity ranging from 91% to 98%.

Cynomolgus macaque peripheral blood (PB) CD34^+^ cells were mobilized with 200 µg/kg G‐CSF and 200 µg/kg SCF on 5 consecutive days. On days 6 and 7, 15–20 ml mobilized PB was collected. CD34^+^ cells were enriched by using anti‐CD34 IgM and MACS IgM microbeads (Miltenyi Biotec) according to the manufacturer’s instructions and as described previously [22, 23]. Enriched CD34^+^ cells were cryopreserved and thawed at indicated time points for culture.

### Cell Culture

In the first stage of culture, 4 × 10^4^ hCB or cynomolgus mobilized PB CD34^+^ cells were seeded in a 24‐well plate in 1 ml of Iscove’s modified Dulbecco’s medium containing putrescine (100 µM), selenium (5 ng/ml), insulin (25 µg/ml), human albumin (1%, vol/vol), transferrin (50 µg/ml), and B‐27 supplements (2%, vol/vol) (referred to as modified IMDM medium) for 6 days upon addition of the CC1 cocktail at 37°C in 5% CO_2_. The CC1 cocktail was composed of SCF (100 ng/ml), Flt‐3L (100 ng/ml), TPO (50 ng/ml), IL‐3 (15 ng/ml), low‐density lipoprotein (LDL; 25 µg/ml; Stem Cell Technologies, Vancouver, BC, Canada, http://www.stemcell.com), and SR1 (1 µM). Fresh medium with cytokines was added on day 3. Subsequently, cells obtained from stage 1 were cultured in a 25‐cm^2^ flask in modified IMDM medium for 7 days and various combinations of cytokines, including SCF (100 ng/ml), TPO (100 ng/ml), IL‐3 (15 ng/ml), LDL (40 µg/ml), IL‐6 (25 ng/ml), IL‐11 (25 ng/ml), GM‐CSF (15 ng/ml), and SR1 (1 µM) (supplemental online Table 1) were screened for the second stage of culture. Fresh medium with cytokines was added every 2 days. At various time points, cells were stained with 0.4% trypan blue and manually counted with a hematocytometer. MKs were identified and quantified with a FACSVerse flow cytometer (BD Biosciences) using the anti‐CD41a and anti‐CD42b antibodies according to the procedure described in Porter et al. [24].

### MK Characterization

At different time points of culture, cells were stained with or without Wright‐Giemsa and observed under an Olympus IX51 microscope (Olympus Corp., Tokyo, Japan, http://www.olympus‐global.com). DNA ploidy was analyzed by flow cytometry, as described previously [25]. Briefly, cells were first incubated with the anti‐CD41a antibody or IgG isotype control in the dark for 15 minutes at room temperature. After incubation, cells were fixed with 1% paraformaldehyde for 1 hour at 4°C and then treated with a trypsin inhibitor/RNase buffer for 10 minutes. Subsequently, cells were stained with propidium iodide for 10 minutes in the dark at 4°C. At least 20,000 cells were analyzed for each sample. CD41a^+^ cells were gated, and at least 20,000 cells were analyzed for each sample. For immunofluorescence staining, cells were incubated with FITC‐conjugated anti‐CD41a antibodies for 1 hour and then washed three times with phosphate‐buffered saline (PBS). Hoechst (10 μg/ml) in PBS was used to stain nuclei for 5 minutes, followed by three more PBS washes and then examined by an Olympus IX51 fluorescence microscope.

### In Vivo Analysis in Mice

After sublethal irradiation (2.5 Gy), NOD/SCID mice were intravenously administered with 1 × 10^7^ hCB‐MK day 6 + 3 cultures (experimental group, *n* = 12) or 100 µl PBS (control group, *n* = 3). PB samples were then collected from the retro‐orbital plexus at different time points after transplantation and stained with human anti‐CD41a and anti‐CD42b antibodies for analysis by flow cytometry. For analyzing human platelet activation, 10 µl PB was collected and incubated with or without ADP (50 µM) at 37°C for 10 minutes, probed with anti‐human CD62P antibody or IgG isotype control, and analyzed by flow cytometry [26, 27]. Mouse bone marrow (BM) was harvested from both femurs, and the expression of human CD45 and CD41a was examined by flow cytometry after red cell lysis [28].

### Transplantation of MKPs or Mature MKs in Nonhuman Primates

Transplantation was performed at least 1 month after cell mobilization and collection procedures. Primates (*n* = 12) were injected intravenously for 3 consecutive days with carboplatin (10 mg/kg per day) for inducing thrombocytopenia. Autologous transplantation of day 6 + 2 MKPs (*n* = 3), and auto‐ and allotransplantations of day 6 + 7 mature MKs (*n* = 5) were performed on days 7 and 15 after carboplatin injection, respectively. As negative controls, primates (*n* = 3) were injected with normal saline; for positive control, a primate (*n* = 1) was transfused with platelets isolated from fresh whole blood. Before transplantation, MKPs were transduced with a lentiviral vector expressing green fluorescent protein (GFP) and mature MKs were labeled with FITC‐microbeads for in vivo detection.

### Cell Labeling

GFP lentivirus was prepared as described [29]. Primate MKPs were transduced with GFP lentiviral particles for 8 hours. Cells were subsequently collected and resuspended in normal saline. Transduction efficiency of GFP lentiviral particles was confirmed by flow cytometry. FITC microbeads were synthesized by conjugating nano‐microspheres with FITC fluorochrome (Lumigenex Co., Ltd., Suzhou, China, http://www.lumigenex.com). Mature MKs were washed and incubated with FITC microbeads for 1 hour at 37°C and then resuspended in normal saline. Labeling efficiency of FITC microbeads was approximately 100%, as confirmed by flow cytometry.

### Hematology

After transplantation, routine whole blood tests were performed on primates by using SysmexXT‐2000iv (Sysmex Corp., Kobe, Japan, http://www.sysmex.com) to monitor hematopoietic cell recovery, including platelet and white blood cell numbers. BM aspiration was performed on day 14 after transplantation, and blood sample smears were fixed and stained with Wright‐Giemsa [30]. GFP^+^ cells and FITC‐fluorescent platelets were detected by flow cytometry [19]. Platelet activation was analyzed by incubating PB with ADP (50 µM) at 37°C for 10 minutes, followed by probe of CD62P expression by flow cytometry. Bleeding time was analyzed by recording the time until the bleeding stopped after a standard cut was made in the forearm of the primates.

### Statistical Analysis

Data were expressed as mean ± SD from three to five independent experiments. Statistical analysis was performed by using the Student’s *t* test, performed with GraphPad Prism, version 5, software (GraphPad Software, Inc., La Jolla, CA, http://www.graphpad.com). A *p* value <.05 was considered to represent a statistically significant difference.

## Results

### Proliferation of hCB CD34^+^ Hematopoietic Stem/Progenitor Cells Ex Vivo in Stage 1 of Culture

**Figure 1:**
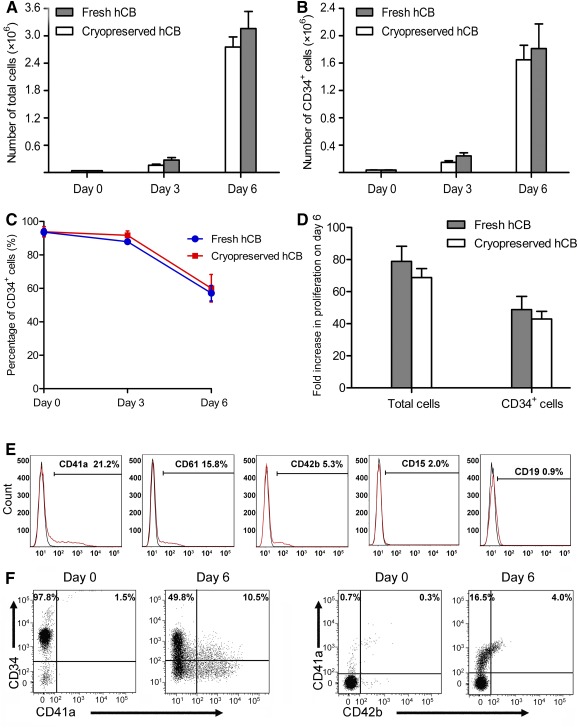
Kinetics of CD34^+^ hematopoietic stem/progenitor cell expansion in stage 1 of culture. **(A, B):** Absolute numbers of total cells and CD34^+^ cells from day 0 to day 6 generated from 4 × 10^4^ fresh or cryopreserved hCB cells in modified Iscove’s modified Dulbecco’s medium supplemented with the CC1 cocktail. Data are mean ± SD; *n* = 3. **(C):** Descending rates of percentage CD34^+^ cells from day 0 to day 6. Data are mean ± SD; *n* = 3. **(D):** Fold‐increase in proliferation of total cells and CD34^+^ cells on day 6. Data are mean ± SD; *n* = 3. **(E):** Representative flow cytometry profiles of CD41a, CD61, CD42b, CD15, and CD19 antigen expression on expanded cells, respectively. (Black line, isotype antibody control; red line, specific antibody). **(F):** Representative dot plots of CD34, CD41a, and CD42b cell‐surface markers on uncultured cells and expanded cells, respectively. Abbreviation: hCB, human cord blood.

We started with both fresh and cryopreserved hCB CD34^+^ cells and evaluated the effects of the cocktail, CC1 (composed of 100 ng/ml SCF, 100 ng/ml Flt‐3L, 50 ng/ml TPO, 15 ng/ml IL‐3, 25 µg/ml LDL, and 1 µM SR1), on ex vivo expansion. During the first 3 days, cells expanded at a slow pace, especially in the cryopreserved hCB group, whereas from day 3 to day 6, the cells cultured with CC1 showed a sharp increase in total and CD34^+^ cells in both groups (Fig. 1A, 1B). After 6 days of culture, there were no significant differences between fresh and cryopreserved hCB cultures in terms of total and CD34^+^ cell numbers and in the percentage of CD34^+^ cells (Fig. 1A–1C). On day 6, CD34^+^ cells in fresh and cryopreserved hCB groups showed a 48‐fold and a 43‐fold increase, respectively (Fig. 1D). These results indicated that CC1 could promote the expansion of both fresh and cryopreserved hCB CD34^+^ cells. Subsequent experiments described were performed with cryopreserved hCB samples because a frozen vial of hCB samples is more available in real‐life clinical situations.

Meanwhile, the expanded cells expressed CD41a, CD61, and CD42b (specific surface markers of the megakaryocytic lineage) at 20.8% ± 3.4%, 15.7% ± 4.6%, and 5.4% ± 1.1%, respectively, and CD15^+^ (granulocyte lineage marker) and CD19^+^ (lymphocyte lineage marker) cells were less than 2%, indicating that differentiation was induced toward the megakaryocytic lineage (Fig. 1E). On day 6, further analysis by coimmunostaining demonstrated that the expanded cells consisted of 10.2% ± 1.8% of CD34^+^/CD41a^+^, 15.9% ± 1.3% of CD41a^+^/CD42b^−^, and 4.0% ± 0.7% of CD41a^+^/CD42b^+^ cells, respectively, which were commonly classified as MKPs (Fig. 1F).

### Induction and Maturation of MKs Ex Vivo in Stage 2 of Culture

**Figure 2:**
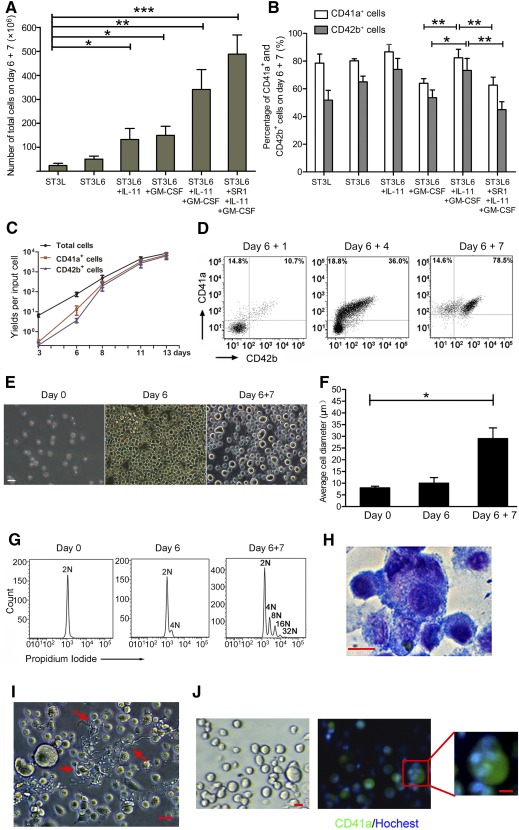
Kinetics, morphology, and characterization analysis of differentiated MKs via two‐stage culture system. **(A,B):** Number of total cells and percentage CD41a^+^ and CD42b^+^ cells from day 6 + 7 cultures induced by the various cytokine cocktails. Data are mean ± SD; *n* = 5. ∗, *p* < .05; ∗∗, *p* < .01; ∗∗∗, *p* < .001. **(C):** Yields of total cells, CD41a^+^ cells, and CD42b^+^ cells per input cryopreserved human cord blood CD34^+^ cell after 6‐day culture with the CC1 cocktail and an additional 7‐day culture with the cocktail CC2. Data are mean ± SD; *n* = 5. **(D):** Representative dot plots of CD41a and CD42b cell‐surface markers during MK differentiation. **(E):** Representative photomicrograph of cells at various stages of expansion and differentiation to MKs. Scale bar = 20 μm. **(F):** Average diameter analysis of cells. Digital images of cells were captured, and ImageJ software (National Institutes of Health, Bethesda, MD, https://imagej.nih.gov/ij/) was used to measure diameters. Data are mean ± SD; *n* > 100. ∗, *p* < .05. **(G):** Representative data of DNA content analysis during MK differentiation. **(H):** Wright‐Giemsa staining of differentiated MKs on day 6 + 7. Scale bar = 20 μm. **(I):** A representative phase contrast image showing the pro‐platelet‐forming MKs (red arrows). Scale bar = 20 μm. **(J):** Immunofluorescence of CD41a (green) marker on the cell surface of MKs. Hoechst (blue) staining shows poly‐nuclei. Scale bars = 20 μm. Abbreviations: GM‐CSF, granulocyte macrophage‐colony‐stimulating factor; IL, interleukin; MKs, megakaryocytes; SR1, StemRegenin 1; ST3L, combination of stem cell factor, thrombopoietin, interleukin‐3, and low‐density lipoprotein; ST3L6, combination of stem cell factor, thrombopoietin, interleukin‐3, low‐density lipoprotein, and interleukin‐6.

Six days after the initial expansion of stage 1, various cytokine combinations were evaluated for the differentiation of mature MKs with high efficiency in stage 2 (Fig. 2A, 2B; supplemental online Table 1). From day 6 + 1 to day 6 + 7, all cytokine cocktails had increases in total, CD41a^+^, and CD42b^+^ cell numbers. Total cell counts after supplementation with ST3L6 (a combination of SCF, TPO, IL‐3, LDL, and IL‐6) + IL‐11 + GM‐CSF and ST3L6 + IL‐11 + GM‐CSF + SR1 cocktails were higher than those in other cytokine cocktails (Fig. 2A). Although ST3L6 + IL‐11 + GM‐CSF + SR1 cocktails induced the highest rate of total cell expansion, the proportions of CD41a^+^ and CD42b^+^ cells were low, suggesting that SR1 had little effect on induction to mature MKs (Fig. 2B). On the basis of both the cell number and the degree of enrichment of induced MKs, the ST3L6 + IL‐11 + GM‐CSF cocktail (called “cocktail CC2” hereafter) was selected to induce differentiation and maturation of MKs in stage 2.

After 6‐day culture with CC1, followed by an additional 7‐day culture with CC2, the normalized yield of MKs was approximately 1.0 × 10^4^ cells from one cryopreserved hCB CD34^+^ cell with 82.4% ± 6.1% of CD41a^+^ and CD42b^+^ cells at 73.3% ± 8.5% (mean ± SD), respectively (Fig. 2B, 2C). The proportions of CD41a^+^CD42b^−^ and CD41a^+^CD42b^+^ cells were analyzed for evaluating the kinetics of MK differentiation and maturation during the culture period (Fig. 2D). Furthermore, morphological changes could be clearly observed after differentiation. Mature MKs were much larger in size than cells on day 0 and day 6 (Fig. 2E). The average cell diameter significantly increased from 8.0 ± 0.7 μm (*n* = 121) on day 0 to 29.1 ± 4.6 μm (*n* = 109) on day 6 + 7 (Fig. 2F). Collectively, these results demonstrate that CC2 may facilitate the differentiation and maturation of human MKs ex vivo.

### Ex Vivo Identification and DNA Ploidy Analysis of Induced MKs

DNA ploidy of induced hCB‐MKs was determined by flow cytometry. CD34^+^ stem/progenitor cells before and after ex vivo expansion were 2N (Fig. 2G). However, DNA ploidy increased to 4N or greater on day 6 + 7, occupying approximately 32.67% ± 7.43% of the total cell population (4N, 19.63% ± 1.81%; 8N, 11.13% ± 1.52%; 8N^+^, 4.00% ± 0.54%). Furthermore, Wright‐Giemsa staining and immunofluorescence analyses revealed characteristic lobular nuclei, numerous α‐granules in the cytoplasm, and expression of CD41a on the cell membrane (Fig. 2H, 2J). In addition, pro‐platelet‐like structures could be observed toward the end of culture, strongly suggesting that some MKs were close to terminal differentiation (Fig. 2I).

### Thrombocytopoiesis Assessment in a Murine Xenotransplantation Model

Next, we determined the platelet release potency of hCB‐MKs in vivo by transplanting day 6 + 3 cultures into NOD/SCID mice that consisted of 20.5% CD34^+^, 53.3% CD41a^+^, 35.6% CD41a^+^/CD42b^+^, and 2.3% CD34^+^/CD41a^+^ cells, respectively.

**Figure 3:**
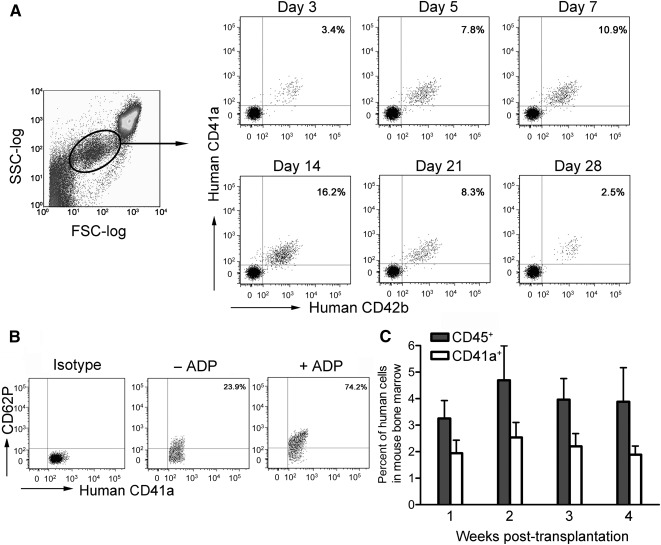
Production of human functional platelets and engraftment in NOD/SCID mice transplanted with differentiated hCB‐MKs. **(A):** Differentiating hCB‐MKs of day 6 + 3 cultures (1 × 10^7^) were transplanted into sublethally irradiated NOD/SCID mice. Human platelets produced were detected in mouse PB at the indicated time points after transplantation. Representative dot plots were visualized according to the FSC‐log and SSC‐log. **(B):** Representative flow cytometry profiles for platelets produced by transplanted hCB‐MKs expressing human CD62P in the presence or absence of ADP (50 µM) on day 14 after transplantation, respectively. Platelet activation is demonstrated by CD62P labeling. **(C):** Human cells engrafted in mouse bone marrow at the indicated time points after transplantation. Data are mean ± SD; *n* = 3 at every time point assayed. Abbreviations: FSC, forward scatter; hCB‐MKs, human cord blood megakaryocytes; SSC, side scatter.

The kinetics of thrombocytopoiesis in the experimental group from day 3 to day 28 after transplantation is shown (Fig. 3A). Three days after transplantation, 3.4% ± 1.8% (mean ± SD, *n* = 3) human platelets were detected in mouse PB and then the platelet proportion gradually increased. Platelet numbers increased between day 7 (10.8% ± 4.3%) and day 14 (13.7% ± 6.2%) and then declined thereafter. Human platelets were not observed in the control group (intravenously injected with PBS) at any time point. Furthermore, human CD62P expression was increased after stimulation by ADP, indicating that platelets released in vivo by transplanted MKs were functional (Fig. 3B). In addition, NOD/SCID mice were sacrificed at weeks 1, 2, 3, and 4 after transplantation for determining short‐term engraftment in mouse BM. Human CD45^+^ cells were detected 1 week after transplantation and the percentage of CD45^+^ cells remained relatively stable during the 4‐week period, indicating successful short‐term engraftment. In addition, human CD41a^+^ MKs were also detected in mouse BM (Fig. 3C).

### Safety Assessment in the Nonhuman Primate Model

To assess the safety and efficacy of MK transplantation for further clinical application, a nonhuman primate model was used. Mobilized PB CD34^+^ cells collected after a G‐CSF/SCF conditioning regimen were cultured by the two‐stage culture system to generate MKs for transplantation because CB samples are not routinely obtained from primates. Thrombocytopenia in primates was induced by intravenous injection of carboplatin at a dose of 10 mg/kg per day for 3 consecutive days. Transplantation of cultured cells revealed no adverse reactions in primates. In particular, no signs of fever, emesis, or diarrhea were observed. Moreover, more than 1 year after transplantation, all primates continued to survive with no apparent abnormalities.

### Hematological Recovery of Primates Transplanted With Autologous MKPs

**Figure 4:**
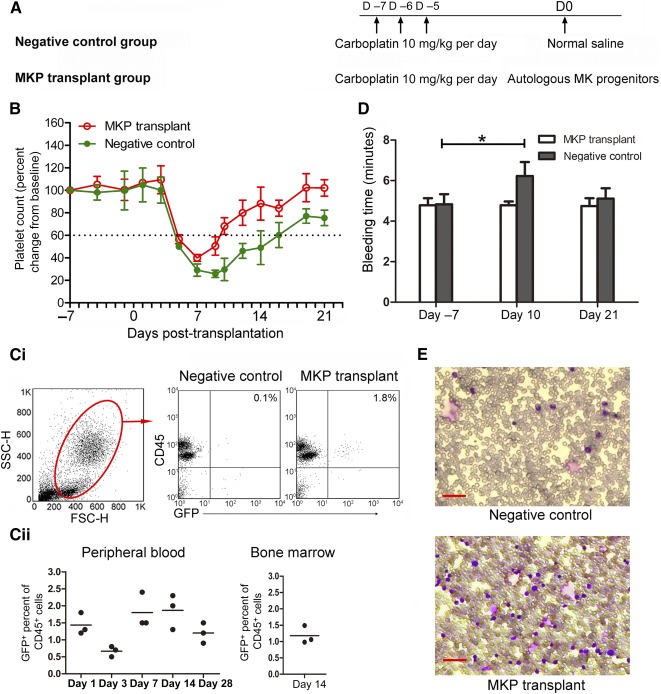
Engraftment kinetics: short‐term hematological recovery in nonhuman primates transplanted with MKPs (day 6 + 2 cultures). **(A):** Scheme for MKP transplantation in a nonhuman primate model. Carboplatin treatment was administered on days −7, −6, and −5, and transplantation was performed on day 0. **(B):** Mean percentage platelet counts from baseline in primates which received MKP preparations (MKP transplantation group) or normal saline (negative control group). The platelet count on day −7 before carboplatin injection was regarded as the baseline. **(C):** MKP preparations that were labeled by GFP were detectable by flow cytometry in primate peripheral blood (PB) and bone marrow (BM) after autologous transplantation. **(Ci):** Representative dot plots of GFP^+^ cells in nucleated cells (CD45^+^ population) from negative control or MKP transplant group. **(Cii):** Percentage GFP^+^ cells among PB and BM nucleated cells in MKP‐transplanted group primates at different time points after transplantation. Lines indicate the median. **(D):** Bleeding time assessment of primates in MKP transplantation group and negative control group at the indicated time points. Data are mean ± SD; *n* = 3. ∗, *p* < .05. **(E):** Fourteen days after transplantation, BM cells of primates were harvested from anterior superior spine and representative BM smears were examined after Wright‐Giemsa staining on an Olympus IX51 microscope (Olympus Corp.). Scale bars = 40 μm. Abbreviations: D, day; FSC, forward scatter; GFP, green fluorescent protein; H, height; MK, megakaryocyte; MKP, megakaryocytic progenitor; SSC, side scatter.

Seven days after carboplatin injection, primates of the MKP transplant group (*n* = 3) were transplanted with induced MKP preparation (day 6 + 2 cultures), whereas primates in the negative control group (*n* = 3) were treated with normal saline (Fig. 4A). The selected cytokine cocktails (CC1 and CC2) were used to expand and induce primate CD34^+^ cells. The results of ex vivo cultivation and composition of transplanted grafts for individual primates are summarized in Table 1. After expansion and differentiation, median count of total cells was 5.14 × 10^7^, which included 50% CD34^+^ cells and 18.5% CD34^+^/CD61^+^ MKPs, respectively. The median cell numbers for transplantation were 1.1 × 10^7^ total cells per kilogram and 2.1 × 10^6^ CD34^+^/CD61^+^ MKPs per kilogram. Platelet counts of the two groups did not change significantly during the first 10 days of carboplatin treatment as compared with the baseline. However, after 10 days, platelet counts declined remarkably in both groups. In the MKP transplant group, the platelet count nadir was 40% of the baseline whereas in the control group, the platelet count nadir was 25% of the baseline. Moreover, 10 days were required for the platelet count to increase to greater than 60% of baseline in the MKP transplant group compared with 16 days required in the control group (Fig. 4B). Therefore, transplantation of MKPs significantly improved platelet count nadir and promoted platelet recovery. Strikingly, white blood cells also recovered rapidly in the MKP transplant group, perhaps because half of the transplanted cells were CD34^+^ stem cells (supplemental online Fig. 1). Carboplatin‐induced myelosuppression in primates also led to a reduced number of leukocytes, and therefore, transplanted CD34^+^ stem cells may differentiate to neutrophils and/or lymphocytes in primates.

**Table 1:** Summary of cellular characteristics and composition of transplanted grafts for individual primates in megakaryocytic progenitor transplant group

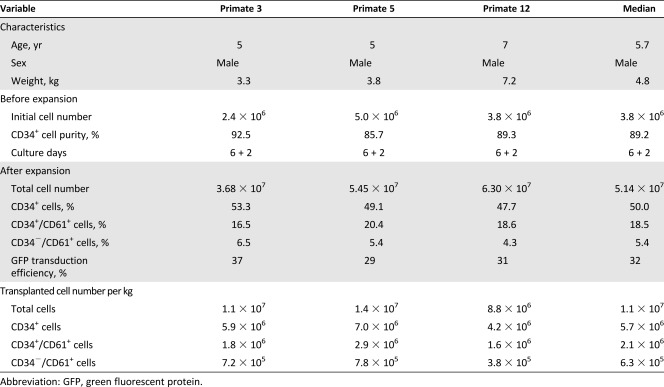

Fluorescent cells could be detected in the PB of MKP transplant group primates up to 28‐day after transplantation, reaching a plateau from days 7 to 14 after transplantation (Fig. 4Ci, 4Cii). In addition, primate BM aspiration also showed approximately 1% of total nucleated cells with fluorescence, indicating successful early engraftment of transplanted cells in primate BM (Fig. 4Cii). Smear examination revealed more active proliferation of nucleated cells in BM of the MKP transplant group (Fig. 4E). Furthermore, to assess the platelet function in vivo, bleeding time was measured. The normal bleeding time for two groups (as positive controls) was approximately 4.5 minutes on day −7 before carboplatin treatment. On day 10, bleeding time of negative control extended for more than 6 minutes because of low platelet counts, with a mean of 30 × 10^9^/liter, whereas bleeding time of the MKP‐transplanted group, with platelet counts of 100 × 10^9^/liter, was similar to that in the positive control group (Fig. 4D). These results indicated that transplantation of MKPs was successful for early engraftment into BM, improving platelet recovery and promoting hemostasis in primates.

### Platelet Recovery for Primates Transplanted With Autologous or Allogeneic Mature MKs

**Figure 5:**
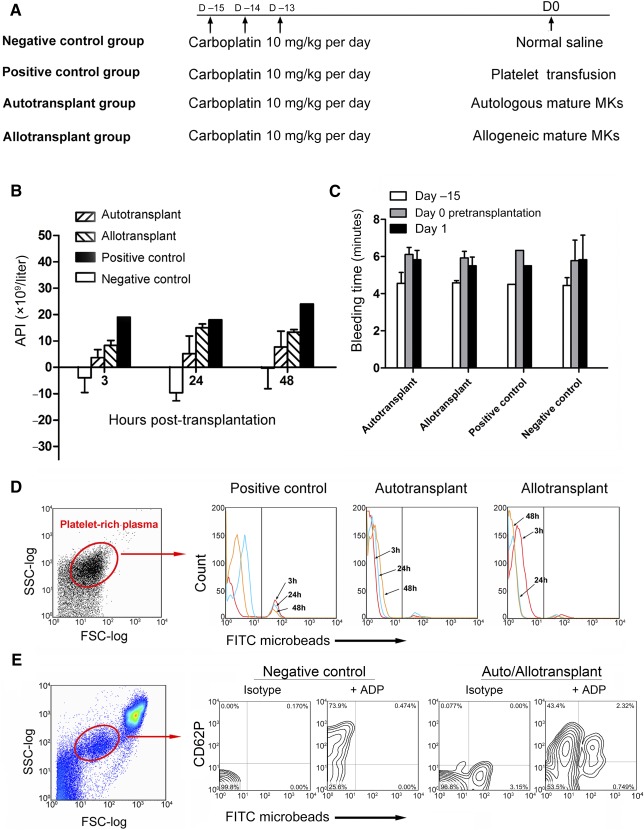
Platelet recovery analysis in nonhuman primates transplanted with autologous or allogeneic mature MKs (day 6 + 7 cultures). **(A):** Scheme for mature MK transplantation in a nonhuman primate model. Carboplatin treatment was administered on days −15, −14, and −13, and transplantation was performed on day 0. **(B):** API analysis of primates in four groups at 3, 24, and 48 hours after transplantation. Data are mean ± SD (negative control group, *n* = 3; positive control group, *n* = 1; autotransplant group, *n* = 3; allotransplant group, *n* = 2). **(C):** Bleeding time assessment of primates in the four groups at different time points. Data are mean ± SD. **(D):** FITC microbeads‐fluorescent platelets were detected by flow cytometry in the PB of primates that received fresh platelets (positive control group), autologous mature MKs (autotransplant group), and allogeneic mature MKs (allotransplant group). Relative flow frequency histograms of fluorescent platelets that were visualized at 3, 24, and 48 hours after transplantation based on FSC‐log and SSC‐log. **(E):** Twenty‐four hours after transplantation, whole PB was incubated with ADP for 10 minutes and the activation of platelets was demonstrated by CD62P labeling. Representative flow cytometry contour plots were visualized according to the FSC‐log and SSC‐log. Abbreviations: API, absolute platelet increment; D, day; FITC, fluorescein isothiocyanate; FSC, forward scatter; MK, megakaryocyte; PB, peripheral blood; SSC, side scatter.

The procedure for mature MK transplantation is described in Figure 5A. On the basis of the results of MKP transplantation, platelet count nadir occurred approximately 15 days after carboplatin injection. Therefore, we chose that day as the optimal time point for transplantation of mature MKs. After expansion and differentiation of primate CD34^+^ cells for 6 + 7 days, total cells were 2.9 × 10^8^, containing 30.3% median proportion of CD61^+^ cells. The mean doses for transplantation were 6.2 × 10^7^ for total cells per kilogram and 1.9 × 10^7^ for CD61^+^ mature MKs per kilogram, respectively (Table 2). The change trend of platelet counts as compared with the baseline is shown in supplemental online Figure 2. In particular, absolute platelet increment (API) was calculated for each group at hours 3, 24, and 48 after transplantation because these were critical time points for evaluating the effect of platelet transfusion in the clinic (Fig. 5B). Three hours after transplantation, API in the positive control (transplanted with freshly isolated platelets) was 19 × 10^9^/liter, and it ranged from 3 × 10^9^/liter to 10 × 10^9^/liter in autotransplant (*n* = 3) and allotransplant (*n* = 2) groups. Twenty‐four to 48 hours after transplantation, API in the positive control remained at a stable level, whereas it continued to increase to 10 × 10^9^/liter and 20 × 10^9^/liter in autotransplant and allotransplant groups, respectively. In addition, flow cytometry detected approximately 1%–3% of fluorescent platelets within 3 hours after transplantation of mature MKs labeled with FITC microbeads in autotransplant and allotransplant groups that were maintained for more than 48 hours. The positive control primate transfused with fresh isolated platelets labeled with FITC microbeads also showed 18% of fluorescent platelets at 3 hours and the proportion gradually decreased to 5.5% at 48 hours (Fig. 5D). Furthermore, the activation of platelets was confirmed by anti‐CD62P staining after ADP stimulation of whole PB, indicating that the platelets released by transplanted mature MKs were functional (Fig. 5E). Furthermore, in MK‐transplanted primates, bleeding times tended to be shorter at day 1 after transplantation as compared with day 0, but these changes were not significant (Fig. 5C).

**Table 2:** Summary of cellular characteristics and composition of transplanted grafts for mature megakaryocyte transplantation

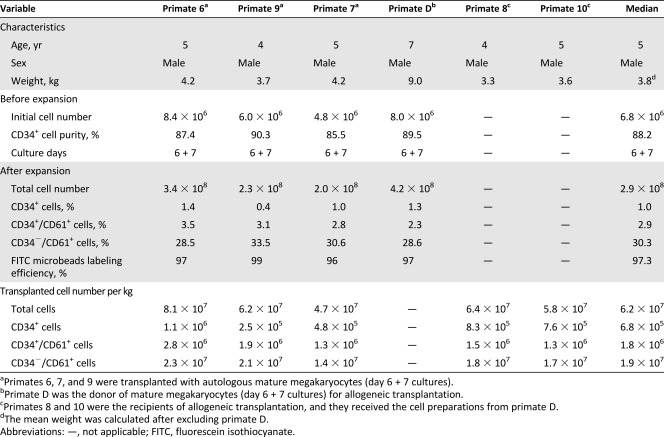

## Discussion

In the current study, we describe a stroma‐free, animal component‐free, and gene manipulation‐independent system for generating MKs from HSCs that is amenable to good manufacturing practice for clinical applications. Through use of this protocol, 1.0 × 10^4^ MKs were obtained from one input cryopreserved hCB CD34^+^ cell, resulting in an approximately 650‐fold higher yield than reported previously [31, 32, 33, 34, 35]. Moreover, platelet counts recovered efficiently without adverse effects in primates that received 2 × 10^6^ MKPs per kilogram or 2 × 10^7^ mature MKs per kilogram. Therefore, the number of ex vivo generated MKPs or mature MKs from one hCB unit (5 × 10^6^ CD34^+^ cells) could, in theory, be used for treating more than 30 patients (with an average weight of 70 kg). In addition, because personalized treatment has been newly introduced in the clinic, transplantation of autologous MKs can also achieve satisfactory therapeutic effects in some patients. Therefore, the effect of two‐stage culture system on the expansion and differentiation of human mobilized PB CD34^+^ cells was also evaluated. Approximately 500 total cells, with CD41a^+^ and CD42b^+^ at 90.4 ± 4.7% and 80.3 ± 7.9%, respectively, were obtained from one human cryopreserved PB CD34^+^ cell (supplemental online Fig. 3).

We have made great effort toward determining optimal cytokine combinations that will promote megakaryocytic differentiation and maturation in the second culture stage. TPO is a major cytokine required for megakaryocytic lineage development, but TPO alone is not sufficient for obtaining large‐scale MK maturation [36]. In this study, IL‐6, in combination with the ST3L cytokine cocktail (combination of SCF, TPO, IL‐3, and LDL), was highly effective in stimulating megakaryopoiesis (Fig. 2A; supplemental online Table 1). IL‐11 also promotes the expansion and maturation of MKPs by modulating synergistic interactions with other early‐acting factors, such as IL‐3 and SCF [36, 37]. The addition of GM‐CSF to the ST3L6 + IL‐11 cocktail increased the expression of CD42b^+^ mature megakaryocytes, perhaps also because of synergistic interactions with IL‐11.

Delayed platelet recovery is a severe obstacle in clinical applications involving hCB transplantation [38]. Previous reports suggested that human circulating platelets appeared in irradiated NOD/SCID mice 10–12 days after transplantation of noncultured CD34^+^ cells [34]. A study by Tijssen and colleagues further implied that predifferentiation of CD34^+^ cells toward the megakaryocytic lineage for at least 7 days was necessary for obtaining rapid in vivo platelet formation [39]. In the current study, human platelet formation was significantly accelerated after transplantation with 6 + 3‐day culture induced hCB‐MKs. Platelets were detected on day 3 and were continuously present for at least 4 weeks in PB circulation in mice. One explanation may be that the cell preparations for transplantation were not homogeneous after ex vivo cell culture. Indeed, 20.5% CD34^+^, 53.3% CD41a^+^, 35.6% CD41a^+^/CD42b^+^, and 2.3% CD34^+^/CD41a^+^ cells were present. Therefore, those human platelets detected as early as 3 days after transplantation were likely to be produced by the more matured CD41a^+^/CD42b^+^ MKs. Subsequently, CD34^+^ stem cells and CD34^+^/CD41a^+^ MKPs engrafted in mouse BM would gradually mature and release platelets in mice, which would persist for at least 4 weeks [40]. CD62P is exposed only on the surface of platelets that can release α‐granules, so the expression of CD62P exhibited the activation state of platelets [41]. Because of the relatively small absolute amount of human platelets released in mice, detection of CD62P after ADP stimulation by flow cytometry is a feasible method to address the platelet function in vivo.

As part of the preclinical evaluation of the safety and efficiency of MK transplantation, we conducted a transplantation protocol in a nonhuman primate model to better mimic clinical situations. Unlike NOD/SCID mice that received human cells with little engraftment rejection, no immunodeficient or immunosuppressed primate model was available. Therefore, nonhuman primate MKs were generated for autologous and allogeneic transplantations. By using the same culture media and cytokine cocktails, the number and the degree of enrichment of MKs derived from mobilized PB CD34^+^ cells of primates were relatively lower than those obtained from hCB CD34^+^ cells. There are two potential explanations for these observations: (a) The intrinsic proliferation potential of hCB CD34^+^ cells is higher than that of PB CD34^+^ cells, and (b) the cytokines used were all recombinant human proteins, which may not fully stimulate primate cells. In addition, culture conditions were not optimized for primate cells because our goal was to validate the feasibility of the protocol and confirm safety and efficacy of the cultured cells in primates. Nonetheless, primate MKs generated by our two‐stage culture system exhibited long‐term safety and efficacy in releasing platelets in vivo, which could provide fundamental guidelines for further phase I clinical trials.

Lentiviral GFP‐labeled primate CD34^+^ cells and MKPs could be traced by flow cytometry and detected 1%–2% of nucleated cells in PB and BM only during the first month after autologous transplantation. It is possible that a part of the transplanted cells differentiated into platelets without nuclei and lost long‐term GFP labels. Additionally, the transduction efficiency of lentiviral GFP was relatively low (less than 30%; Table 1) and exogenously labeled GFP^+^ cells may have been gradually diluted out as the recipient’s own hematopoietic cells recovered.

Previous studies showed that culture‐derived MKs were functional and released platelets on complete maturation [42, 43]. However, a common problem underlying the various protocols detailing platelet production ex vivo is the low yield of platelets, with only 6–30 platelets obtained from one MK [11, 12, 44]. In our study, the CC2 cocktail increased the proportion of MKs with a ploidy level of 8N or greater, which may be differentiated to produce more platelets because of the bloodstream shear force in vivo [45]. Therefore, the number of platelets released per MK can be increased to 50–100 (calculated by Fig. 5B). More importantly, for clinical use, transfusion of platelets with the same blood ABO typing is mandated because of existing antibodies in platelet preparations. In our studies that did not involve blood cross‐typing and HLA matching, acute and chronic graft‐versus‐host disease were not observed in primates even a year after receipt of the allogeneic mature MKs. Therefore, blood typing may not be essential for ex vivo culture‐derived MKs because the entire cell culture process is plasma‐ and antibody‐free. Collectively, transfusion of megakaryocytes is a promising therapy method that would alleviate platelet shortage problem; in addition, the data presented herein will serve as a basis for comparing new therapies in the future.

## Conclusion

We have developed an efficient protocol for the production of MKs and platelets, which can be explored for transplantation to alleviate thrombocytopenia. Importantly, our autologous and allogeneic transplantation studies in nonhuman primates strongly suggest that the induced MKs may be safe and efficacious in potential clinical applications.

## Author Contributions

X.G. and M.Q.: conception and design, collection and/or assembly of data, data analysis and interpretation, manuscript writing, final approval of manuscript; Y.Z. and B.S.: collection and/or assembly of data, data analysis and interpretation, manuscript writing; Y.W., Z.R., and X.D.: provision of study material or patients, data analysis and interpretation; W.D. and Y.J.: conception and design, financial support, manuscript writing, final approval of manuscript.

## Disclosures of Potential Conflicts of Interest

M.Q. and Z.R. are employees of Biopharmagen Corp. Y.J. has a leadership position with Biopharmagen Corp. The other authors indicated no potential conflicts of interest.

## Supporting information

Supporting Information
