# Supplementary material for: Safety and Efficacy of Megakaryocytes Induced from Hematopoietic Stem Cells in Murine and Nonhuman Primate Models
Source: Stem Cells Transl Med. 2016 Oct 7;6(3):897–909. doi: 10.5966/sctm.2016-0224 (PMC5442772; doi:10.5966/sctm.2016-0224)
Supplement: Supplementary file 1 — Supporting Information [file SCT3-6-0897-s001.pdf]

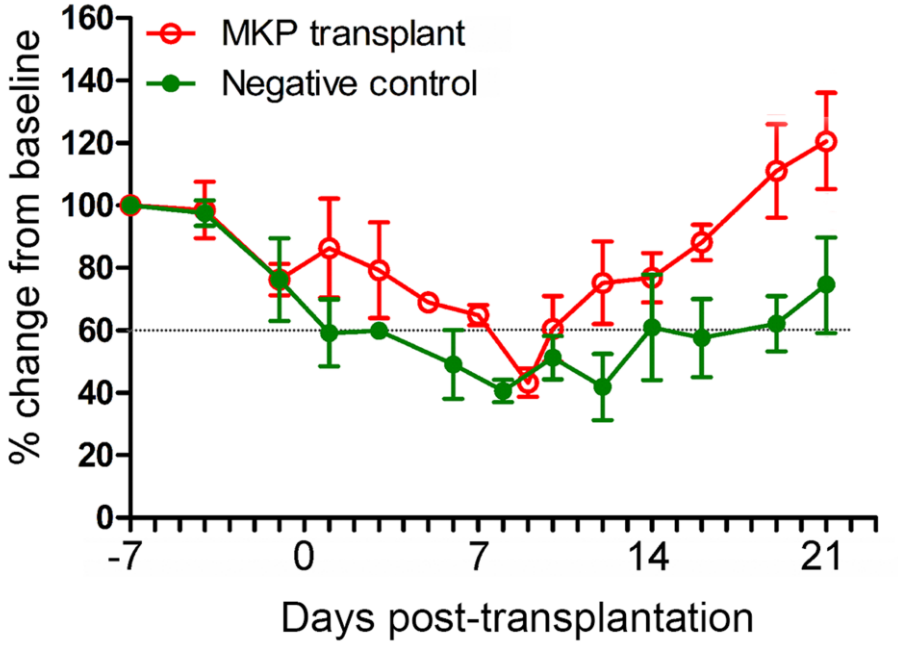


**Figure S1.** Mean percentage change of white blood cell counts from baseline. Carboplatin treatment (10mg/kg/d) was performed on days -7, -6, and -5 and transplantation of day 6+2 MKPs (MKP transplantation group) or normal saline (Negative control group) was performed on day 0. The platelet count on day -7 before carboplatin injection was regarded as the baseline.

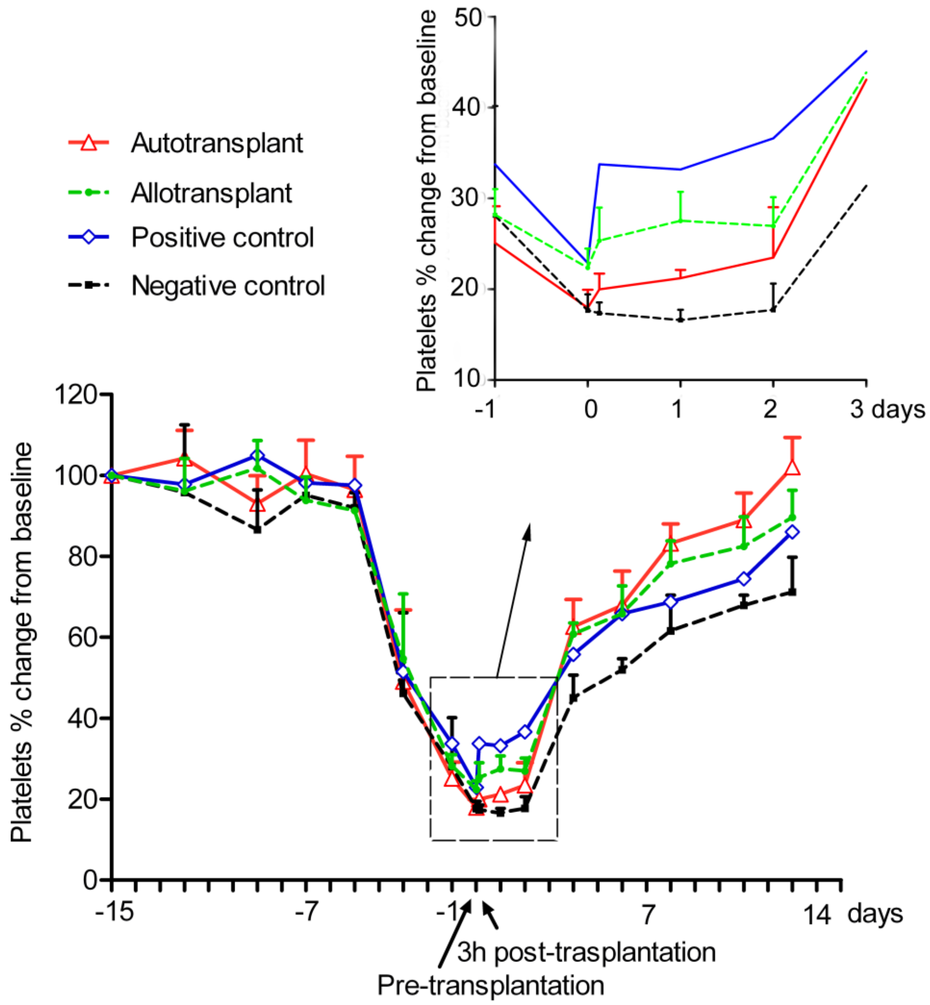


**Figure S2.** Mean percentage change of platelet counts from baseline. Carboplatin treatment (10mg/kg/d) was performed on days -15, -14, and -13 and transplantation of autologous or allogeneic 6+7-day culture induced mature MKs were performed on day 0. Meanwhile, negative control primates received normal saline and positive control primates received platelets that isolated from fresh whole blood. The platelet count on day -15 before carboplatin injection was regarded as the baseline.

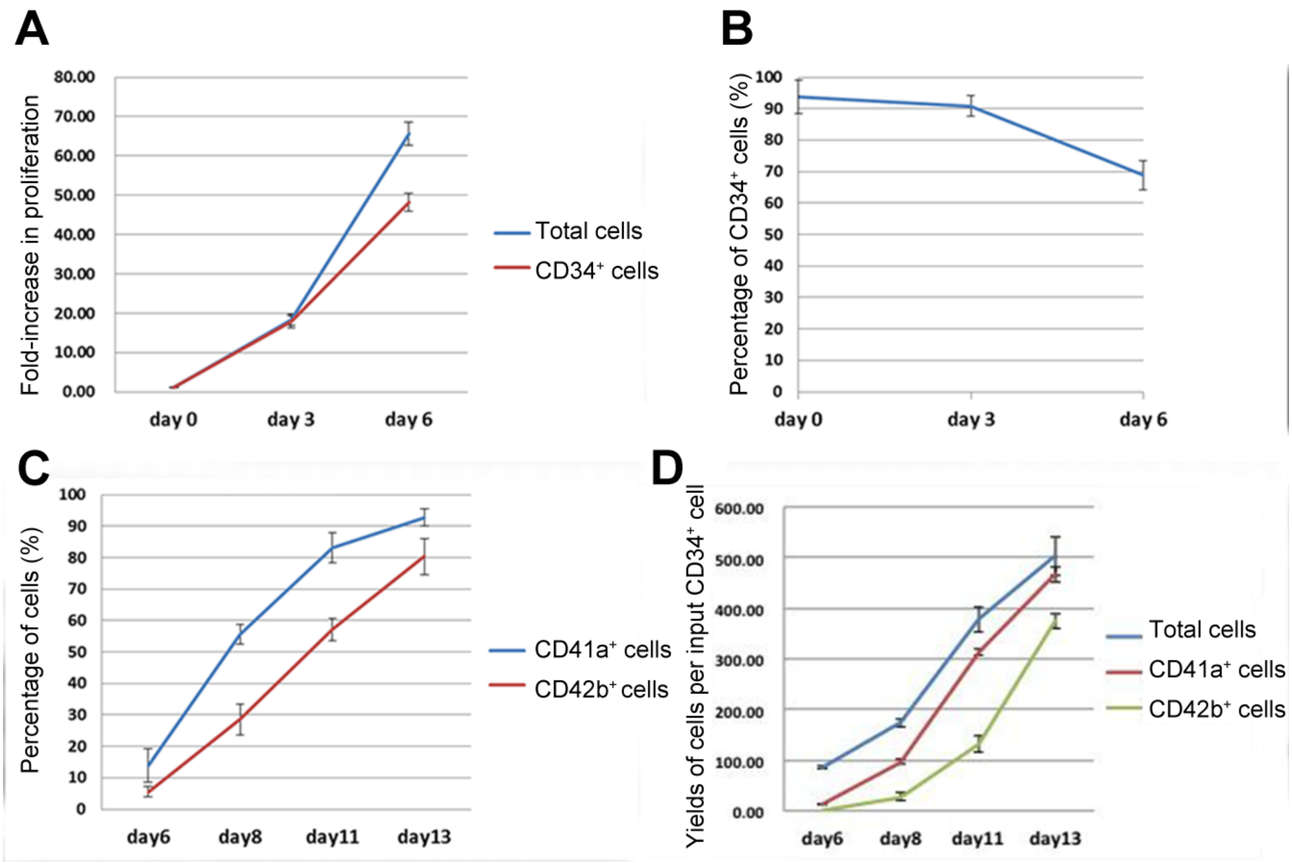


**Figure S3.** Expansion and differentiation of human PB CD34$^+$ cells via two-stage culture system. (A) Proliferation folds of total cells and CD34$^+$ cells from day 0 to day 6 generated by cryopreserved PB CD34$^+$ cells in modified IMDM medium supplemented with the CC1 cocktail. (B) Descending rates of percentage of CD34$^+$ cells from day 0 to day 6. (C) Percentage of CD41a$^+$ and CD42b$^+$ cells in stage 2 stimulated by CC2 cocktail. (D) Yields of total cells, CD41a$^+$ cells and CD42b$^+$ cells per input cryopreserved PB CD34$^+$ cell by optimized two-stage culture system. Data are mean ± SD; n = 3.

**Supplemental Table**

**Table S1.** Induction of megakaryocytes with various cytokine combinations in stage 2 of culture

| | **Absolut number of cells on day 6+7 (× $10^4$)** | | | **Percent of cells on day 6+7 (%)** | |
|---|---|---|---|---|---|
| | **Total** | **$CD41a^+$** | **$CD42b^+$** | **$CD41a^+$** | **$CD42b^+$** |
| ST3L | 2377 ± 955.2 | 1873 ± 790.9 | 1263 ± 626.7 | 78.5± 6.6 | 51.8 ± 7.1 |
| ST3L6 | 5100 ± 1233.9 | 4145 ± 902.9 | 3344± 971.9 | 80.2 ± 1.5 | 65.0 ± 4.1 |
| ST3L6+IL-11 | 13268 ± 4569.9 | 116879 ± 4688.9 | 10080 ± 4416.4 | 86.7± 5.3 | 74.0 ± 7.9 |
| ST3L6+GM-CSF | 14968 ± 3794.9 | 9619 ± 2727.9 | 8075 ± 2466.7 | 64.0 ± 3.3 | 53.6 ± 5.6 |
| ST3L6+IL-11+GM-CSF | 34124 ± 8261.6 | 28474 ± 9026.7 | 25454 ± 89927.9 | 82.4 ± 6.1 | 73.3 ± 8.6 |
| ST3L6+IL-11+GM-CSF+SR1 | 48814 ± 8043.6 | 30721 ± 7049.7 | 22113 ± 5579.1 | 62.6 ± 5.8 | 44.9 ±5.7 |

Data are shown as mean ±SD of 5 independent experiments.

Abbreviations: ST3L, a cytokine cocktail including stem cell factor, thrombopoietin, interleukin-3 and low density lipoprotein; ST3L6, a cytokine cocktail including stem cell factor, thrombopoietin, interleukin-3, low density lipoprotein and interleukin-6; IL-11, interleukin-11;

GM-CSF, granulocyte-macrophage colony-stimulating factor; SR1, stem regenin 1.
